# [^18^F]SynVest-1 PET imaging in people with Parkinson’s disease

**DOI:** 10.1093/braincomms/fcaf258

**Published:** 2025-07-16

**Authors:** Sarah L Martin, Carme Uribe, Kimberly L Desmond, Lucas Narciso, Bayla Dolman, Edgardo Torres-Carmona, Isabelle Boileau, Ariel Graff-Guerrero, Neil Vasdev, Antonio P Strafella

**Affiliations:** Temerty Faculty of Medicine, University of Toronto, 27 King's College Circle, Toronto, Ontario M5S 1A1, Canada; Brain Health Imaging Centre, Centre for Addiction and Mental Health (CAMH), Toronto M5T 1R8, Canada; Department of Psychology, Faculty of Health and Education, Translational and Computational Neuroscience Unit, Manchester Metropolitan University, Manchester M15 6GX, UK; Brain Health Imaging Centre, Centre for Addiction and Mental Health (CAMH), Toronto M5T 1R8, Canada; Krembil Brain Institute, University Health Network, University of Toronto, Toronto, Ontario M5T 1S8, Canada; Brain Health Imaging Centre, Centre for Addiction and Mental Health (CAMH), Toronto M5T 1R8, Canada; Department of Psychiatry, University of Toronto, Toronto M5T 1R8, Canada; Brain Health Imaging Centre, Centre for Addiction and Mental Health (CAMH), Toronto M5T 1R8, Canada; Department of Psychiatry, University of Toronto, Toronto M5T 1R8, Canada; Brain Health Imaging Centre, Centre for Addiction and Mental Health (CAMH), Toronto M5T 1R8, Canada; Brain Health Imaging Centre, Centre for Addiction and Mental Health (CAMH), Toronto M5T 1R8, Canada; Department of Psychiatry, University of Toronto, Toronto M5T 1R8, Canada; Brain Health Imaging Centre, Centre for Addiction and Mental Health (CAMH), Toronto M5T 1R8, Canada; Department of Psychiatry, University of Toronto, Toronto M5T 1R8, Canada; Brain Health Imaging Centre, Centre for Addiction and Mental Health (CAMH), Toronto M5T 1R8, Canada; Department of Psychiatry, University of Toronto, Toronto M5T 1R8, Canada; Brain Health Imaging Centre, Centre for Addiction and Mental Health (CAMH), Toronto M5T 1R8, Canada; Department of Psychiatry, University of Toronto, Toronto M5T 1R8, Canada; Temerty Faculty of Medicine, University of Toronto, 27 King's College Circle, Toronto, Ontario M5S 1A1, Canada; Brain Health Imaging Centre, Centre for Addiction and Mental Health (CAMH), Toronto M5T 1R8, Canada; Krembil Brain Institute, University Health Network, University of Toronto, Toronto, Ontario M5T 1S8, Canada; Neurology Division, Department of Medicine, Morton and Gloria Shulman Movement Disorder Unit and Edmond J. Safra Program in Parkinson Disease, Toronto Western Hospital, University Health Network, University of Toronto, Toronto M5T 2S6, Canada

**Keywords:** synaptic density, Parkinson’s disease, PET, synaptic vesicle glycoprotein 2A, SV2A, [^18^F]SynVest-1

## Abstract

The [^18^F]SynVest-1 radiotracer targets the synaptic vesicle glycoprotein 2A (SV2A) and is a proxy of presynaptic density. Parkinson’s disease is associated with synaptic dysfunction. Here we investigated synaptic density via the [^18^F]SynVest-1 radiotracer in people with PD compared with healthy controls, with reference to how it compares to the previous SV2A radiotracer, [11C]UCB-J. Ten Parkinson’s patients and 12 healthy subjects underwent a [^18^F]SynVest-1 PET scan. We compared non-displaceable binding potential via voxel-wise and volume of interest analysis to investigate group differences. Volume-of-interest-analyses reported lower non-displaceable binding potential in key a priori regions associated with Parkinson’s disease, namely the substantia nigra and caudate nucleus (*P* < 0.05). Follow-up exploratory volume-of-interest-analyses reported widespread reduction in non-displaceable binding potential within all brain lobes, cerebellum, hippocampus, thalamus and insula; however, these findings did not survive correction for multiple comparisons (*P* < 0.004). In addition, voxel-wise analyses with family-wise error correction, highlighted significantly lower non-displaceable binding potential in the PD cohort within the putamen and cerebellum. We did not observe any relationships between clinical metrics and non-displaceable binding potential. The results are in line with differences observed using the [^11^C]UCB-J radiotracer. The [^18^F]SynVest-1 radiotracer confirmed lower synaptic density in the Parkinson’s disease cohort and adds to the growing evidence of synaptic dysfunction in Parkinson’s disease pathology.

## Teaser

Martin *et al*. report that the radiotracer [18F]SynVest-1 reveals reduced synaptic density in Parkinson’s disease compared with healthy controls, particularly in the substantia nigra, putamen, and caudate. Exploratory analyses suggest widespread reductions, highlighting synaptic dysfunction as a hallmark of Parkinson’s pathology and supporting prior findings with [11C]UCB-J.

## Graphical Abstract

**Graphical Abstract:**
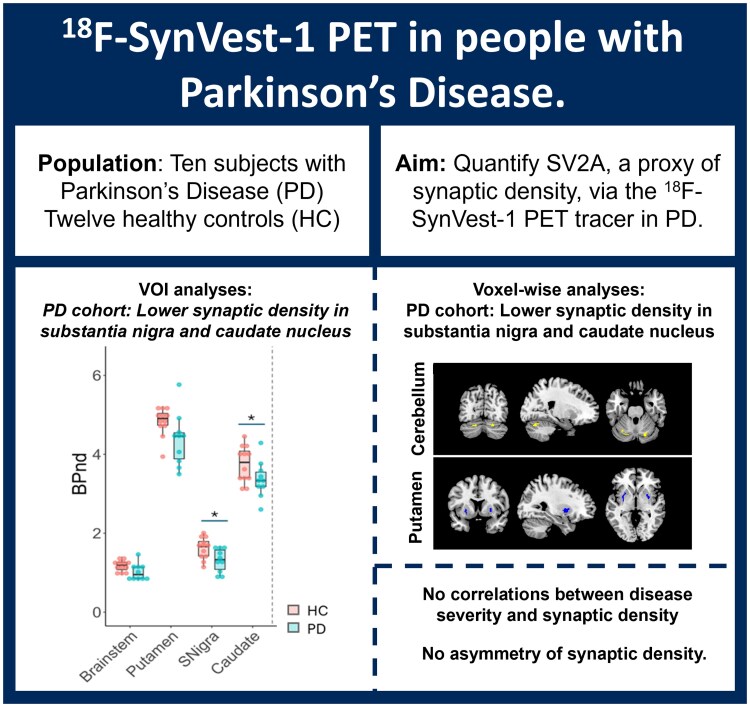

## Introduction

There is growing evidence that synaptic dysfunction and neuronal damage occurs in Parkinson's disease (PD). For instance, many of the genes associated with PD, such as α-synuclein, LRRK2, DJ-1, PINK1, and PRKN, are critical for presynaptic function, and knockout mouse models have revealed that the loss of these genes can disrupt synaptic plasticity and neurotransmitter release.^1-7^ Dysfunctional vesicular dynamics within the synapse have also been implicated in PD pathology.^6-9^ Although often characterized by dopaminergic neuron loss, many neurotransmitter systems are involved in the pathology of the disease, indicating widespread neurodegeneration. One hypothesis of PD progression is that degeneration begins within the lower brainstem, progressing up to the midbrain, then later stages of degeneration occurring in the cortex of the brain.^10^ Therefore, temporal and spatial patterns of synaptic density changes may help track the progression of PD.

**Figure 1:**
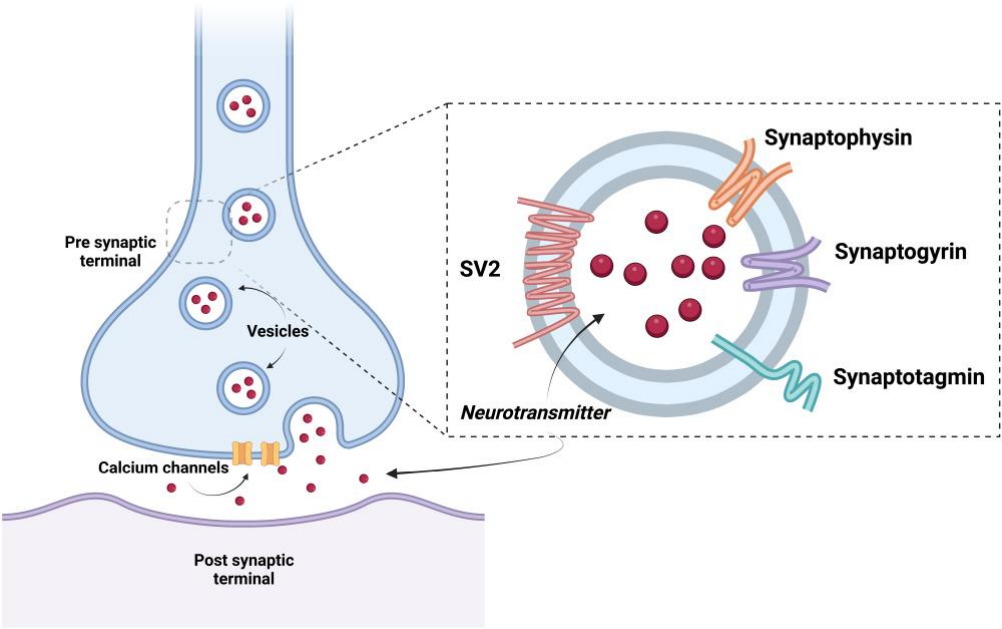
**A simplified schematic diagram of the location of synaptic vesicle (SV2) proteins.** The SV2 proteins are on the membrane of vesicles located in the presynaptic terminal. The SV2A isoform are ubiquitously expressed, whereas the SV2-B and -C isoforms are less widespread. The vesicle membranes also contain other proteins (including Synaptophysin, Synaptogyrin, and Synaptotagmin) which amongst others regulate the release of neurotransmitters across the synapse. SV2 is known to be involved in the regulation of calcium-dependent exocytosis. To note, the imaging of synaptophysin has been regarded to be the ‘gold-standard’ of in vitro quantification of synaptic density. Hence, quantification of SV2 via radiotracer binding in vivo and Synaptophysin in vitro are closely correlated. Figure generated with BioRender (https://BioRender.com/r60×820), originally published in Martin *et al*.^27^

Given the significant role of synaptic and neuronal changes in the pathology of PD, tools capable of measuring synaptic density *in vivo* are crucial for understanding the disease. The positron emission tomography (PET) radiotracers [^11^C]UCB-J and [^18^F]SynVest-1 target the presynaptic vesicular protein SV2A and are a proxy of presynaptic density (see Fig. 1).^11^ The SV2A isoform of the SV2 protein is expressed in all grey matter with varying expression levels.^12-14^ whereas the SV2-B and -C isoforms are less widespread. Therefore, targeting SV2A is a good candidate for *in vivo* synaptic density. Not only SV2A is important for the regulation of neurotransmitter release and other roles associated with synaptic function,^14^ but it has been shown to interact with PD-related genes, potentially leading to impaired vesicle transport and neuronal dysfunction.

The earlier form of the SV2A radiotracer, [^11^C]UCB-J, has highlighted reduced synaptic density in PD-related brain regions in people with PD. Previous research has shown lower SV2A in the substantia nigra, brain stem nuclei and other midbrain and cortical regions.^15-17^ While Delva *et al*.^16^ and Wilson *et al*.^17^ found no correlations between clinical metrics and SV2A abundance, Holmes *et al*.^18^ concluded that reduced synaptic density in the substantia nigra was associated with greater disease severity. Therefore, *in vivo* measurement of SV2A via PET has revealed PD-related brain changes and holds promise for diagnostic and disease progression applications. Due to its better pharmacokinetics and longer half-life, [^18^F]SynVest-1 will be used as the synaptic density marker in future studies; therefore, replication of [^11^C]UCB-J findings is vital to allow comparisons across studies using different methodologies.^19^

Here we use the [^18^F]SynVest-1 radiotracer to investigate differences between PD and a healthy cohort for the first time.

## Materials and methods

### Ethical approval

The study was conducted under the guidelines of the Centre for Addiction and Mental Health (CAMH) Research Ethics Board (REB) and Health Canada regulations, including the Tri-Council Policy Statement of Ethical Conduct for Research Involving Humans. Consent was obtained according to the Declaration of Helsinki. All sessions were performed at CAMH Brain Health Imaging Centre PET and magnetic resonance imaging (MRI) facilities.

### Participants

Ten individuals with PD and twelve healthy controls were included in this study. Participants were excluded if they had any current or prior clinically significant medical or neurological conditions (apart from PD) that might influence the study results. Additional exclusion criteria included, current pregnancy (verified by pregnancy tests at both the screening visit and on the day of PET imaging), breastfeeding, or any contraindications to MRI. PD diagnosis was confirmed via a movement disorders neurologist.

### Assessments

Disease severity was assessed via the movement disorders society unified PD rating scale (MDS-UPDRS) and H&Y by a movement disorders neurologist in the medication ‘ON’, state. Motor function severity was determined via MDS-UPDRS part-III. Cognitive status was assessed via the Montreal cognitive assessment (MoCA) and participants with score < 26 were deemed to have MCI. Seven of the 12 HC participants did not complete a MoCA but were deemed cognitively normal via the Cognitive National Institutes of Health (NIH) battery. Medications were noted for all participants and the Levodopa equivalent daily dose was calculated (reported in Supplementary Table 1).

### Image acquisition

The synthesis of [^18^F]SynVesT-1 was as previously described by Li *et al*.^20^ with modifications by Desmond and colleagues.^21^ PET scans were obtained on a GE Discovery MI 5-ring PET/computed tomography (CT) scanner. Movement of participants was limited using a thermoplastic face mask (Tru-Scan Imaging, Annapolis, USA). A CT scan was acquired for attenuation correction. The PET scan duration was either 120 min or 90 min. Smart *et al*.^22^ has confirmed that a 90-min scan with no arterial line was suitable for the [^18^F]SynVest-1 tracer quantification in a subgroup of our PD cohort.^22^ Dynamic emission data were acquired in list mode beginning 30 s prior to radiotracer injection. 192 ± 10 MBq of [^18^F]SynVesT-1 was administered via bolus injection to the antecubital vein (HC, 195 ± 7 MBq; PD, 188 ± 12 MBq). T_1_-weighted images were acquired using a 3-Tesla GE Discovery MR750 scanner at a separate session.

### PET processing

Data processing was carried out in PMOD (version 4.2, Zurich, Switzerland). PMOD toolboxes were used to preprocess the data. Interframe motion was corrected for by realigning frames to a midpoint frame. Motion correction transformation matrix was created from non-attenuated data and applied to the attenuation correction dynamic PET image. PET images were aligned to the subject’s T_1_-weighted anatomical MRI. Each anatomical image was normalized and resliced to the Montreal Neurological Institute (MNI; McGill University, Montreal, Canada) template and a probabilistic segmentation map was created using the Hammers atlas to define grey matter volumes of interest (VOIs).^23^ The centrum semiovale (CS) VOI was created as described in Rossano *et al*.^24^ and Smart *et al*.^22^ and used as a reference region due to low SV2A binding.^22^ The VOIs were transformed into the subject PET space and refined to only include grey matter voxels (or white matter voxels for CS VOI). Time activity curves (TACs) were extracted for 0–90 min within each of the VOIs of the dynamic PET image. Non-displaceable binding potential *BP*_ND_ was calculated using the simplified reference tissue model 2 (SRTM2), using the CS region as reference. A group average of $k_{2}^{\prime }$ of 0.0415 min^−1^ was used in the calculation of *BP*_ND_. Individual VOIs were extracted and combined to form larger VOIs (i.e. frontal, temporal, parietal, and occipital lobes, as well as the basal ganglia). To account for differences in volume across regions, a volume-weighted average of BP_ND_ was calculated.

### Statistical analyses

Statistical analyses were conducted in RStudio (version 2023.03.0) and statistical parametric mapping (version 12, SPM12; https://www.fil.ion.ucl.ac.uk/spm/software/spm12/) in MATLAB (version R2023b). Age and sex were compared between PD and HC cohorts using independent *t*-test and Chi-squared test, respectively. *BP*_ND_ were compared both at VOI and voxel levels. Group differences, controlling for age and sex, were assessed using multivariate analyses with *post hoc* tests. VOI analyses were conducted on *a priori* selected regions associated with early PD pathology, namely substantia nigra, brainstem, caudate, and putamen.^18^ Exploratory analyses investigated frontal, temporal, parietal, and occipital lobes, as well as basal ganglia, thalamus, cerebellum, pallidum, nucleus accumbens, hippocampus, and insula. Initial analyses used bilateral *BP*_ND_ values. Due to the asymmetric presentation of PD,^25,26^ follow-up analyses of hemispheric differences and most affected side (MAS) versus least affected side (LAS) in the PD cohort were conducted via multivariate analyses. MAS was defined using the MDS-UPDRS-III score and the contralateral VOIs selected for MAS, and ipsilateral for LAS. In addition, group differences in CS volume were calculated to establish potential influence on *BP*_ND_ calculation. Relationships between VOIs and clinical metrics in the PD cohort were assessed using Spearman rank. Voxel-wise analyses were conducted in SPM12. For this, *BP*_ND_ brain maps were transformed into MNI space and included into an ANOVA model. Parameters were set as independent groups, unequal variance, no grand mean scaling or normalisation, age and sex as covariates. Glass brain results were restricted to GM regions and clusters size > 20. Significant differences were identified if they met a voxel-level threshold of < 0.001 and survived FWE-correction at the cluster level (<0.05). FWE correction adjusts *P*-values to account for multiple comparisons and ensures overall probability of false positive (Type I error) is below *P* < 0.05.

Primary and exploratory results are reported as uncorrected (*P* < 0.05), with a statement highlighting whether the exploratory results survived Bonferroni correction for multiple comparisons (*P* < 0.004). Average results are presented alongside its standard deviation (SD).

## Results

### Demographics

Table 1 reports the summary of demographic and clinical characteristics. The PD cohort (mean age, 69 ± 7 years) was significantly older than the HC cohort (56 ± 9 years), (*t* = −3.77, *P* = 0.001). Therefore, all subsequent analyses comparing groups included age as a covariate. Due to the high odds ratio (3.4), sex was included in all statistical modelling as a covariate. The MoCA score ranged from 16 to 30, with 2 subjects being classified as MCI (score = 16 and 22). Whilst MoCA scores did not significantly differ between groups, *t* = 2.14, *P* = 0.058, this was a comparison of only 5 HC scores and 10 PD scores and may not accurately reflect group differences in cognition. No group difference was observed in injected dose of [^18^F]SynVest-1 (*t* = 1.41, *P* = 0.174). Within the PD cohort, disease duration ranged from 4 to 15 years, total MDS-UPDRS score from 33 to 87, and the H&Y score from 2 to 3. No significant group difference was observed in CS (reference region) [HC: 1.77 (0.24) cm^3^, PD: 1.96 (0.48) cm^3^, *P* = 0.207, including age and sex as covariates, mean (SD)].

**Table 1:** Demographic and clinical characteristics

Group	HC (*n* = 12)	PD (*n* = 10)	*P*-value
Sex (F/M)	9/3	4/6	0.192
Age (years)	56 (9)	69 (7)	0.001
MoCA (/30)^a^	29 (1)	26 (4)	0.058
H&Y (/5)	NA	2.4 (0.4)	-
MDS-UPDRS-I (/16)	NA	9.3 (3.3)	-
MDS-UPDRS-II (/52)	NA	9.6 (3.7)	-
MDS-UPDRS-III (/108)	NA	31.4 (11.8)	-
MDS-UPDRS-IV (/44)	NA	2.6 (2.7)	-
MDS-UPDRS Total (/176)	NA	53.1 (17.1)	-
Disease duration (years)	NA	9 (5)	-
Injected dose (MBq)	194 (7)	188 (12)	0.174

Data presented as mean (SD).

F, female; H&Y, Hoehn and Yahr Scale; HC, healthy controls; M, male; MBq, megabecquerel; MDS-UPDRS, movement disorders society unified Parkinson’s disease rating scale; MoCA, Montreal Cognitive Assessment; PD, Parkinson’s disease; SD, standard deviation.

^a^MoCA statistical analysis included 5 HC scores and 10 PD scores.

### Group differences in *BP*_ND_

**Figure 2:**
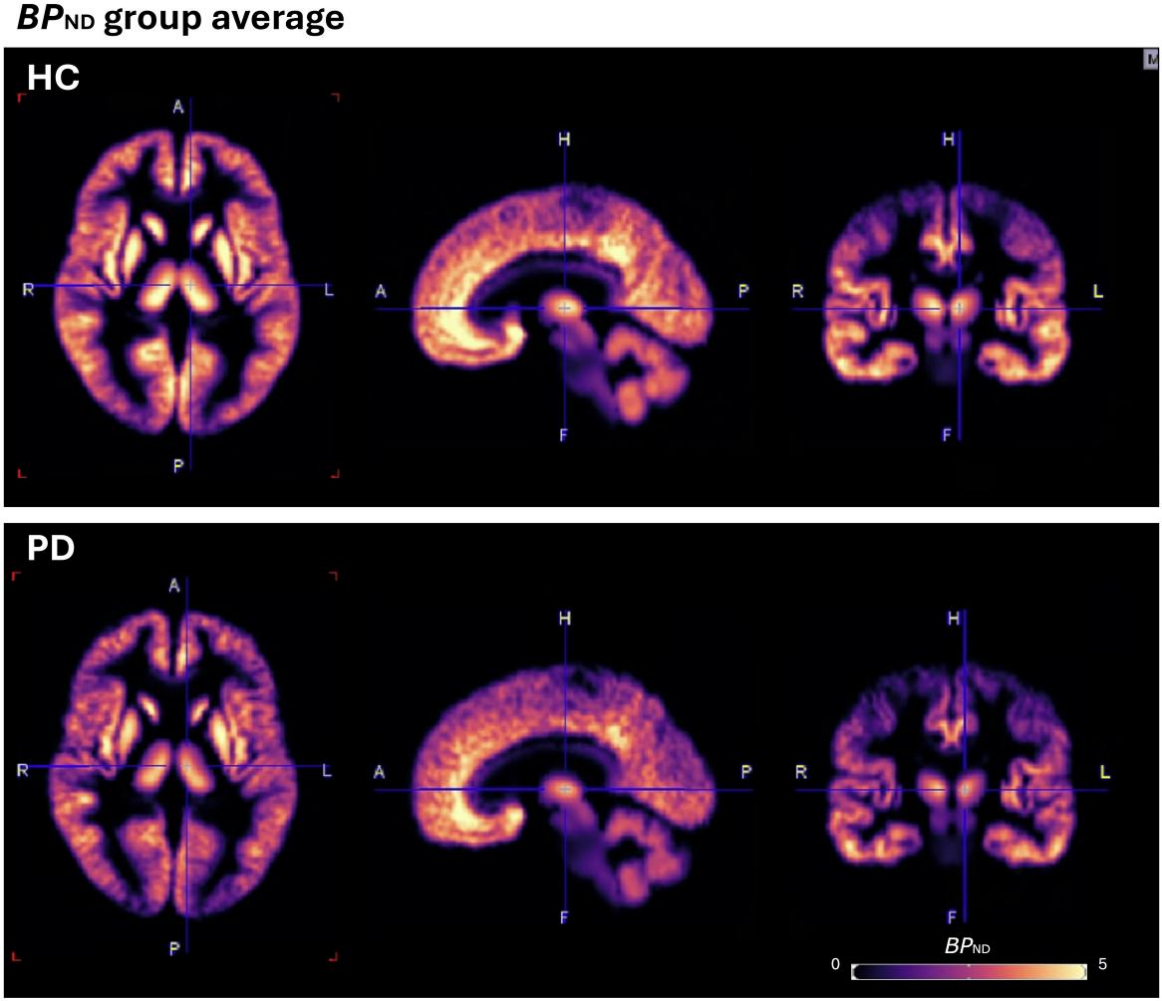
**Grouped average of BP_ND_ brain maps (HC: *n* = 12, PD: *n* = 10).** Figure centred on MNI *x*:49, *y*:55, *z*:40. Colour scale represents BP_ND_. Visual inspection shows lower BP_ND_ in PD cohort compared with healthy controls.

Visual inspection of the BP_ND_ brain maps highlight lower BP_ND_ in the PD cohort compared with HC (Fig. 2).

#### Voxel-wise based group differences in BP_ND_

**Figure 3:**
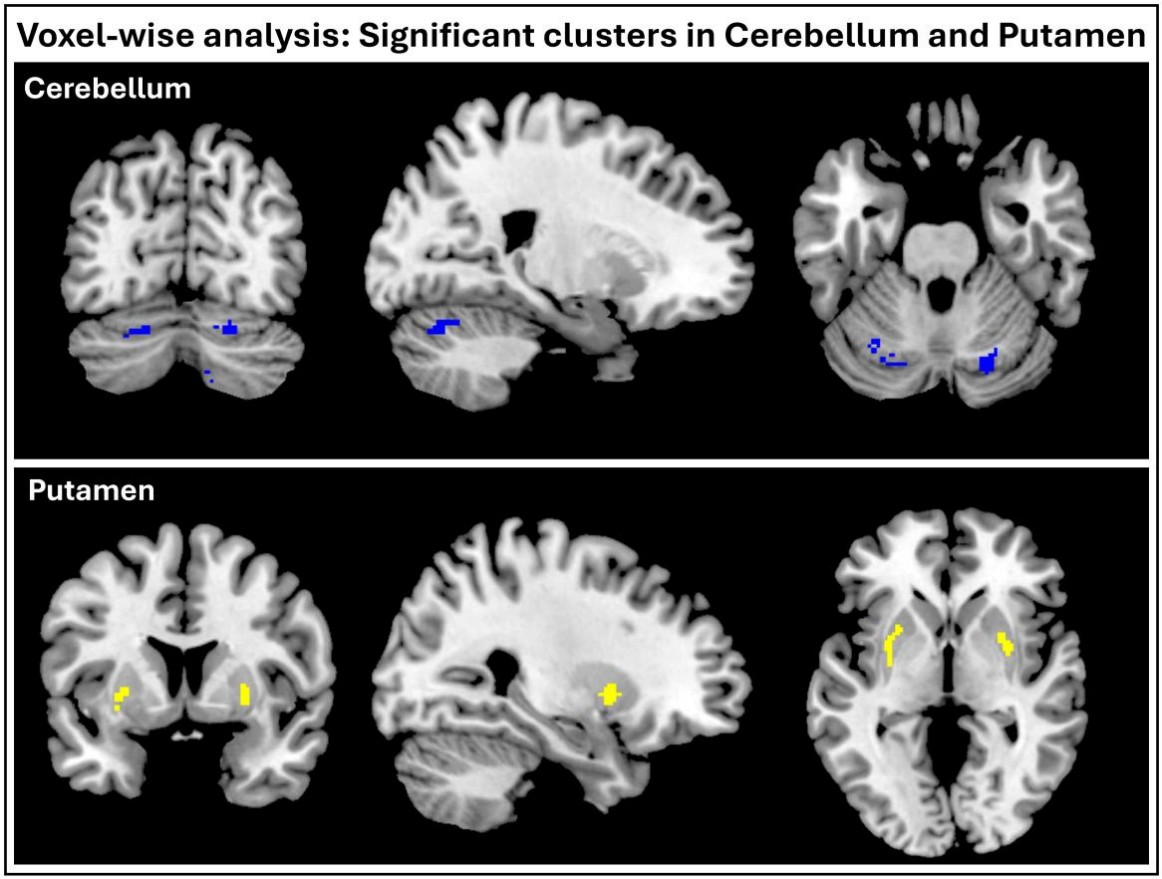
**Significant clusters from whole brain voxel-wise analysis of HC versus PD cohort (HC: *n* = 12 versus PD: *n* = 10).** Cerebellum is centred on MNI [21 −72 −26], and Putamen is centred on [28 8 −2]. Cerebellum clusters are the combination of all five cerebellum clusters reported in Table 2. Clusters are overlayed on MNI space T1-weighted image for visualisation. Significant differences were identified if they met a voxel-level threshold of < 0.001 and survived FWE-correction at the cluster level (<0.05). FWE correction adjusts *P*-values to account for multiple comparisons and ensures overall probability of false positive (Type I error) is below *P* < 0.05.

Whole brain voxel-wise analyses highlighted lower *BP*_ND_ in the putamen and cerebellum. Significant clusters are reported in Table 2 and shown in Fig. 3. Regions survived FWE correction.

**Table 2:** Summary of voxel-wise group differences in BP_ND_

VOI	Cluster #	MNI	K_E_	*p*-uncorr	*p*-FWE	VOI Overlap (%)
Right cerebellum	1	18 −80 −48	57	< 0.001	< 0.001	71%
	2	20 −70 −24	76	< 0.001	< 0.001	92%
Left cerebellum	1	−40 −60 −52	29	< 0.001	< 0.001	85%
	2	−30 −66 −26	30	< 0.001	< 0.001	91%
	3	−36 −56 −54	20	0.003	0.005	87%
Right putamen	1	32 3 −4	59	< 0.001	< 0.001	36%
Left putamen	1	−26 10 0	61	< 0.001	< 0.001	51%

Regions were labelled using Hammers atlas and percent cluster overlaps calculated.

K_E_, number of contiguous voxels; MNI, Montreal Neurological Institute; p-FWE, family wise error *P*-value; p-uncorr, uncorrected *P*-value; VOI, volume of interest.

#### VOI based group differences in BP_ND_

**Figure 4:**
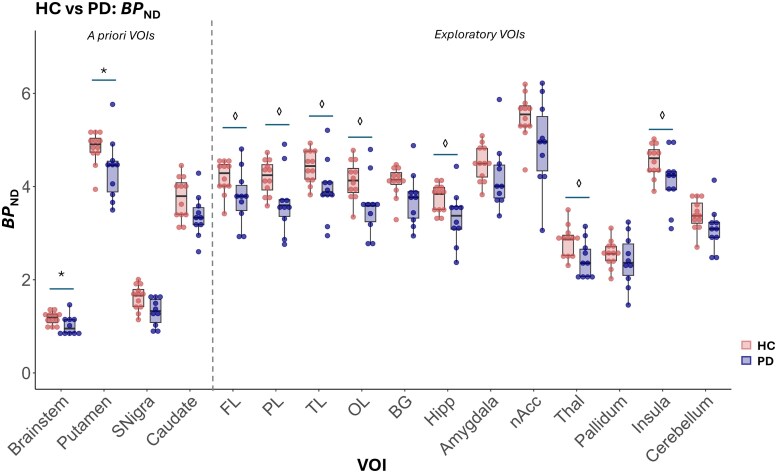
**Summary plot of BP_ND_ in all VOIs in HC and PD cohorts.** Significantly lower BP_ND_ was observed in the substantia nigra and caudate nucleus in the PD cohort compared with the controls. Although lower BP_ND_ was observed in exploratory VOIs of the PD cohort, no exploratory VOIs survived correction for multiple comparisons (*P* < 0.004). SNigra, substantia nigra; FL, frontal lobe; PL, parietal lobe; TL, temporal lobe; OL, occipital lobe; BG, basal ganglia; Hipp, hippocampus; nAcc, nucleus accumbens; Thal, thalamus; VOIs, volumes of interest; HC, healthy controls; PD, Parkinson’s disease; BP_ND_, non-displaceable binding potential. * Denotes a significant difference between groups when assessed via ANOVA with age and sex as covariates (uncorrected, *P* < 0.05). For exploratory VOIs, ◊ denotes a significant difference at uncorrected threshold (<0.05), that did not survive Bonferroni correction (< 0.004).

Table 3 and Fig. 4 reports significantly lower *BP*_ND_ in the *a priori* regions substantia nigra and caudate. Lower *BP*_ND_ was seen in exploratory regions, namely frontal, parietal, temporal, and occipital lobes, as well as hippocampus, thalamus, and insula. However, exploratory VOIs did not survive correction for multiple comparisons (*P* > 0.004).

**Table 3:** Mean BP_ND_ values across groups and ANOVA results

Brain Region	*BP*_ND_		Difference (%)	*F*-value	*P*-value
	HC (*n* = 12)	PD (*n* = 10)			
Brainstem	1.2 (0.1)	1.0 (0.2)	−13.6%	4.03	0.060
Putamen	4.8 (0.4)	4.4 (0.7)	−10.1%	4.21	0.055
Substantia Nigra	1.6 (0.3)	1.3 (0.3)	−21.2%	6.51	0.020^a^
Caudate	3.7 (0.5)	3.4 (0.5)	−10.7%	4.43	0.050^a^
Frontal lobe	4.2 (0.4)	3.8 (0.6)	−10.7%	4.66	0.045^b^
Parietal lobe	4.2 (0.4)	3.7 (0.7)	−13.8%	6.53	0.020^b^
Temporal lobe	4.4 (0.4)	3.9 (0.6)	−11.5%	5.13	0.036^b^
Occipital lobe	4.1 (0.4)	3.6 (0.6)	−14.3%	6.72	0.018^b^
Basal ganglia	4.1 (0.3)	3.7 (0.6)	−9.7%	3.90	0.064
Hippocampus	3.8 (0.3)	3.3 (0.6)	−11.8%	4.85	0.041^b^
Amygdala	4.5 (0.4)	4.2 (0.7)	−6.6%	1.45	0.244
Nucleus Accumbens	5.5 (0.5)	4.9 (0.9)	−11.3%	3.84	0.066
Thalamus	2.8 (0.3)	2.4 (0.4)	−15.2%	8.46	0.009^b^
Pallidum	2.6 (0.3)	2.4 (0.6)	−6.1%	0.65	0.432
Insula	4.6 (0.3)	4.1 (0.6)	−9.8%	4.77	0.042^b^
Cerebellum	3.4 (0.3)	3.1 (0.5)	−9.2%	3.11	0.095

Data presented as mean (SD). All statistical testing included age and sex as covariates. A priori regions and exploratory regions are separated by horizontal line. *P*-value threshold for a priori is < 0.05 and exploratory < 0.004 (Bonferroni corrected). Percent difference between HC and PD average BP_ND_ values is presented.

HC, healthy control; PD, Parkinson’s disease; SD, standard deviation.

^a^Denotes significant difference (*P* < 0.05).

^b^Denotes a significant difference at uncorrected threshold (< 0.05), that did not survive Bonferroni correction (< 0.004).

#### Correlations in PD population: BP_ND_ versus disease duration, UPDRS, and MoCA

We failed to detect any significant correlations between *BP*_ND_ and disease duration, MDS-UPDRS (total/parts I-IV), H&Y, or MoCA score, with either VOI or voxel-wise analysis. Supplementary Table 2 summarizes the Spearman rank correlation coefficients (*r_s_*) for VOI analyses.

#### Asymmetry of BP_ND_

Multivariate analyses using all VOIs reported no hemispheric asymmetry in *BP*_ND_. No significant effect of hemisphere was reported in the full cohort [*F*(1,3) = 0.80, *P* = 0.437], nor was there a significant interaction between group and hemisphere [*F*(1,3) = 0.68, *P* = 0.468]. This indicates that *BP*_ND_ is symmetrical across hemispheres of HC and PD subjects. In the PD cohort, no significant difference was observed between MAS versus LAS [*F*(1,3) = 0.10, *P* < 0.754].

## Discussion

The [^18^F]SynVest-1 radiotracer successfully measured a reduction of SV2A in individuals with PD. These findings are in line with previous conclusions drawn using the [^11^C]UCB-J radiotracer and shows comparability between both radiotracers.^15-18^ Building on previous research that supports the use of SV2A as a proxy of synaptic density,^11,27^ we provide further evidence that synaptic dysfunction is a feature of PD pathology.

The *a priori* regions (caudate, putamen, substantia nigra, and brainstem) were selected due to their involvement in the early stages of the disease and were predicted a more pronounced difference in the PD brain. We observed a reduction in synaptic density within the PD cohort in the substantia nigra, caudate and putamen, however, we did not observe changes in the brainstem. Future research focusing on a more detailed segmentation of brainstem nuclei which could help detect region-specific changes. Lower synaptic density in the putamen was only reported using voxel-wise, and not VOI-analyses. This may be explained by the finer spatial resolution of voxel-wise analysis identifying regional differences within the putamen. We observed lower synaptic density in exploratory regions including all brain lobes, hippocampus, thalamus, insula, and cerebellum. Despite lower synaptic density within individual VOIs of the basal ganglia, there was no overall difference observed in the basal ganglia VOI. This may be due to no differences seen in the nucleus accumbens and pallidum VOIs. However, it’s important to note that no differences observed in the exploratory VOIs survived correction for multiple comparisons.

Our findings of lower synaptic density in the substantia nigra, caudate and putamen are in line with PD pathology and studies using the ^11^C-UCB-J radiotracer.^15-17^ A significant reduction of dopaminergic transmission occurs in the putamen and caudate of people with PD, which is potentially associated with synaptic dysfunction.^28-32^ In post-mortem PD brains, reduced density, length and complexity of dendrites within the caudate and putamen have been noted.^33^

The reduction of SV2A in the cerebellum seen via voxel-wise analysis was only seen in a small cluster, and thus caution must be taken when interpreting these results. Dysfunction of the cerebellum is associated with both motor and non-motor symptoms of PD and thus is not an unexpected finding.^34,35^ For instance, atrophy of the cerebellum occurs in PD, including the degeneration of the cortico-ponto-cerebellar pathway—a key pathway for processing current and proceeding movement, especially smooth motor activity.^34,36,37^ To the best of our knowledge, synaptic changes have not been reported in the cerebellum of people with PD. However, SV2A quantification via [^18^F]SDM-16 radioligand in post-mortem essential tremor patients showed ∼50% reduction in synaptic density in the cerebellar cortex compared with controls.^38^

We did not observe any relationships between synaptic density and clinical metrics. These findings align with those of Delva *et al*.^16^ and Wilson *et al*.^17^ who used the [^11^C]UCB-J radiotracer in drug-*naïve* or early-stage PD patients. However, Holmes *et al*.^18^ reported that reduced synaptic density in the substantia nigra was associated with poorer motor function in PD. The discrepancy between studies may stem from differences in sample sizes and patient heterogeneity. Delva *et al*.^16^ and Wilson *et al*.^17^ focused on drug-*naïve* and/or early-stage PD cohorts, potentially limiting the detection of relationships between disease severity and SV2A abundance. Future research should include a broader range of disease severity to further investigate these connections and determine whether a causal relationship exists.

This study's small sample size is a notable limitation. Despite this, it successfully demonstrated that the [^18^F]SynVest-1 tracer was comparable to the [^11^C]UCB-J tracer in a cohort of individuals with PD. Previous assessments, such as those by Naganawa *et al*.^19^ have shown that [^18^F]SynVesT-1 tends to have higher *BP*_ND_ values than [^11^C]UCB-J (although not statistically significant).^19^ This trend was observed when comparing our results to other studies in PD using the [^11^C]UCB-J radiotracer.^15,18^ Further studies using [^18^F]SynVest-1 in larger cohorts and more varied disease stages of PD, will confirm synaptic density changes in PD. A larger sample size will be able to investigate differences in synaptic density between PD-subtypes (i.e. tremor or gait impaired dominate PD, or PD-MCI, PDD cases), and relationships with medication or treatment response. An additional limitation is the notable age difference between cohorts, requiring age to be included as a covariate across all analyses. It is important to note that wide-spread reduction in synaptic density (assessed via [^11^C]UCB-J or [^18^F]SynVest-1) is not prominent during aging.^39,40^ However, the caudate does appear to be sensitive to aging and has previously shown a ∼1.7% reduction per decade.^40^

Lastly, we must acknowledge that SV2A tracer binding being used as a proxy of synaptic density is based on the assumption that the numbers of vesicles and SV2A expression are not altered by PD pathology. The changes observed may also be identifying a lower abundance of vesicles within the presynaptic terminal, or a change in expression level per vesicle. Although, the number of SV2A proteins per vesicle is considered to be relatively consistent across vesicles, all studies have been conducted in healthy rodent models.^41-44^ Vesicular dysfunction is a well-known pathology in people with PD and thus, we cannot assume that vesicular number or SV2A expression remains consistent compared with healthy aging.^7,8,45^ It could be more appropriate to characterize the observed differences in SV2A as reflecting synaptic changes or dysfunction, rather than density. The spatial and temporal pattern of SV2A could help to explain PD pathology, distinguish between atypical Parkinsonism’s, and could act as a therapeutic biomarker. To conclude, further research is required to determine whether the spatial and temporal pattern of SV2A changes could help to explain PD pathology, distinguish between PD-subtypes and/or atypical Parkinsonism, and could act as a therapeutic biomarker. Alongside post-mortem and pre-clinical research, the SynVest-1 radiotracer will be a vital tool in the future of this research.

## Supplementary Material

fcaf258_Supplementary_Data

## Data Availability

Raw and processed data is available by reaching out to corresponding author. As imaging processing was conducted in PMOD software, no analysis scripts are available to be shared.
