# Supplementary material for: [18F]SynVest-1 PET imaging in people with Parkinson’s disease
Source: Brain Commun. 2025 Jul 16;7(4):fcaf258. doi: 10.1093/braincomms/fcaf258 (PMC12264488; doi:10.1093/braincomms/fcaf258)
Supplement: fcaf258_Supplementary_Data [file fcaf258_supplementary_data.pdf]

Supplementary Table 1: Medication and Levodopa equivalent dose

Subject	Medication	LEDD
1	Levocarb, Mirapex, Entacapone	1098
2	Levocarb, Pramipexole	375
3	Levocarb, Pramipexole, Amantadine	1425
4	Levocarb, pramipexole	550
5	Levocarb, Levocarb CR	1225
6	Levocarb, Parmipexole	800
7	Levocarb	300
8	Levocarb, Mirapex	308
9	Levocarb, Levocarb CR	525
0	Levocarb, Amantadine, Pramipexole	1275

CR; Controlled release, LEDD; Levodopa equivalent daily dose

Supplementary Table 2: Spearman Rank Coefficients for PD group

ROI	UPDRS-total	UPDRS-I	UPDRS-II	UPDRS-III	UPDRS-IV	H&Y	Disease Duration	MoCA
Brainstem	0.15	0.27	0.15	0.09	-0.30	-0.14	0.09	0.14
Putamen	0.09	0.17	0.21	-0.03	-0.24	-0.01	0.10	0.30
Substantia nigra	-0.02	0.18	-0.10	-0.03	-0.43	-0.51	-0.35	0.26
Caudate nucleus	-0.04	-0.04	0.07	-0.10	-0.24	0.06	0.12	0.41
Frontal lobe	-0.01	-0.02	0.14	-0.12	-0.14	0.14	0.18	0.46
Parietal lobe	-0.11	-0.16	0.07	-0.18	-0.24	0.04	-0.11	0.67
Temporal lobe	-0.10	-0.07	0.08	-0.20	-0.02	0.26	0.26	0.52
Occipital lobe	-0.27	-0.35	-0.20	-0.20	-0.39	-0.07	-0.15	0.58
Basal ganglia	0.05	0.10	0.15	-0.05	-0.21	0.03	0.15	0.30
Hippocampus	-0.18	-0.06	-0.08	-0.25	-0.25	0.01	0.14	0.32
Amygdala	0.18	0.09	0.31	-0.02	0.09	0.28	0.41	0.22
Nucleus Accumbens	-0.02	-0.12	0.02	0.03	-0.32	0.01	0.01	0.45
Thalamus	-0.27	-0.24	-0.19	-0.24	-0.45	-0.13	-0.12	0.46
Pallidum	0.11	0.36	0.18	-0.03	-0.36	-0.32	-0.15	0.19
Insula	-0.24	-0.19	-0.09	-0.31	-0.17	0.13	0.17	0.47
Cerebellum	-0.13	-0.11	0.04	-0.16	-0.02	0.32	0.28	0.50

Spearman Rank, r_s coefficient reported for all pairs. (BP_{ND} Parietal Lobe vs MoCA, $p = 0.034$, but did not survive correction for multiple comparisons ($p < 0.004$)).
